# AIDS defining opportunistic infections in patients with high CD4 counts in the combination antiretroviral therapy (cART) era: things ain't what they used to be

**DOI:** 10.7448/IAS.17.4.19621

**Published:** 2014-11-02

**Authors:** Valentin Gisler, David Kraus, Johannes Nemeth, Marthe Than Lecompte, Laurent Merz, Marcel Stoeckle, Patrick Schmid, Enos Bernasconi, Rainer Weber, Matthias Cavassini, Alexandra Calmy, Luigia Elzi, Hansjakob Furrer

**Affiliations:** 1 Department of Infectious Diseases, Bern University Hospital and University of Bern, Bern, Switzerland; 2 Department of Infectious Diseases and ISPM, Bern University Hospital and University of Bern, Bern, Switzerland; 3 Department of Infectious Diseases, University Hospital Zurich, Zurich, Switzerland; 4 Department of Infectious Diseases, University Hospital Geneva, Geneva, Switzerland; 5 Department of Infectious Diseases, University Hospital Lausanne, Lausanne, Switzerland; 6 Department of Infectious Diseases, University Hospital Basel, Basel, Switzerland; 7 Infectious Diseases, Cantonal Hospital St Gall, St Gall, Switzerland; 8 Infectious Diseases, Ospedale Civico Lugano, Lugano, Switzerland; 9 Department of Infectious Diseases, University Hospital Basel, Basel, Switzerland

## Abstract

**Introduction:**

According to reports from observational databases, classic AIDS-defining opportunistic infections (ADOIs) occur in patients with CD4 counts above 500/µL on and off cART. Adjudication of these events is usually not performed. However, ADOIs are often used as endpoints, for example, in analyses on when to start cART.

**Materials and Methods:**

In the database, Swiss HIV Cohort Study (SHCS) database, we identified 91 cases of ADOIs that occurred from 1996 onwards in patients with the nearest CD4 count >500/µL. Cases of tuberculosis and recurrent bacterial pneumonia were excluded as they also occur in non-immunocompromised patients. Chart review was performed in 82 cases, and in 50 cases we identified CD4 counts within six months before until one month after ADOI and had chart review material to allow an in-depth review. In these 50 cases, we assessed whether (1) the ADOI fulfilled the SHCS diagnostic criteria (www.shcs.ch), and (2) HIV infection with CD4 >500/µL was the main immune-compromising condition to cause the ADOI. Adjudication of cases was done by two experienced clinicians who had to agree on the interpretation.

**Results:**

More than 13,000 participants were followed in SHCS in the period of interest. Twenty-four (48%) of the chart-reviewed 50 patients with ADOI and CD4 >500/µL had an HIV RNA <400 copies/mL at the time of ADOI. In the 50 cases, candida oesophagitis was the most frequent ADOI in 30 patients (60%) followed by pneumocystis pneumonia and chronic ulcerative HSV disease (Table 1). Overall chronic HIV infection with a CD4 count >500/µL was the likely explanation for the ADOI in only seven cases (14%). Other reasons (Table 1) were ADOIs occurring during primary HIV infection in 5 (10%) cases, unmasking IRIS in 1 (2%) case, chronic HIV infection with CD4 counts <500/µL near the ADOI in 13 (26%) cases, diagnosis not according to SHCS diagnostic criteria in 7 (14%) cases and most importantly other additional immune-compromising conditions such as immunosuppressive drugs in 14 (34%).

**Conclusions:**

In patients with CD4 counts >500/ µL, chronic HIV infection is the cause of ADOIs in only a minority of cases. Other immuno-compromising conditions are more likely explanations in one-third of the patients, especially in cases of candida oesophagitis. ADOIs in HIV patients with high CD4 counts should be used as endpoints only with much caution in studies based on observational databases.

**Table 1:** Cases of ADOIs occurring in patients with CD4 counts >500/**µ**L according the SHCS database (Total) and the most likely explanation for these OIs according to an in-depth chart review. Chronic HIV infection is the most likely explanation in only a minority of cases

Opportunistic Infection	Total	Chronic HIV with CD4 >500	Unmasking IRIS	CD4 <500	Primary HIV	Other immuno-deficiency	Wrong diagnosis
Candida esophagitis	30	2	0	6	5	15	2
Pneumocystis	9	1	0	6	0	1	1
chronic ulcerative HSV	4	0	0	0	0	1	3
Mycobacterium avium complex (MAC)	2	1	1	0	0	0	0
Progressive multifocal leukoencephalopathy (PML)	2	1	0	0	0	0	0
Cytomegalovirus (CMV)	2	1	0	0	0	0	1
Cryptosporidiosis	1	1	0	0	0	0	0
Total	50	7	1	0	5	17	7

